# The Effects of Nintendo Wii Fit on Postural Balance Control Training in the Geriatric Population: A Review

**DOI:** 10.7759/cureus.31420

**Published:** 2022-11-12

**Authors:** Anushka Raipure, Pooja Kasatwar

**Affiliations:** 1 Department of Community Health, Ravi Nair Physiotherapy College, Datta Meghe Institute of Medical Sciences, Wardha, IND

**Keywords:** nintendo wii fit, physical therapy, geriatric population, postural balance control training, virtual reality, nintendo wii

## Abstract

Elderly populations who engage in consistent, moderate-intensity exercise are more physically active and have better health than elders who have a sedentary lifestyle. Ageing causes the quality and number of muscle fibres to decline, leaving them with less endurance and ability to effectively work in synchronization and less strength and stamina to support weight or keep a sound body system. Medical practitioners often use the word "balance" across many therapeutic disciplines. Balance is commonly used in conjunction with other ideas like stability and postural control. The capacity of the physiological systems to keep the centre of mass within the base of support during static and dynamic postures and to govern physical alignment in an upright position is known as "balance." Impaired balance has a wide range of effects that can harm physical functionality. Falls in the elderly can result in hip fractures and significant morbidity and mortality. Balance dysfunction is one of the primary reasons behind decreased mobility and postural control in the elderly. It mainly affects the ability to walk and to maintain balance control in everyday life. Virtual reality (VR) can be defined as an interaction model between humans and computers that allows conventional computer-based training. It is a novel and expanding technology combining various characteristics like interactivity, independence, and rehabilitation training. These technologies replicate a seemingly natural environment. A fun and engaging aspect of VR technology is the Wii Fit games. These are enjoyable versions of the fundamental exercise and are widely used. A physiotherapy intervention programme can be structured using Wii Fit games to perform balance exercises. These games are widely used for balance training in the geriatric population.

## Introduction and background

There will likely be over two billion seniors (those over 60) by 2050, with Asia being home to most of them. Each year, 37.3 million falls occur worldwide, most affecting those 65 and older [1]. Elderly populations who engage in consistent, moderate-intensity exercise are more physically active and have better health than elders who have a sedentary lifestyle [2]. Ageing causes the quality and number of muscle fibres to decline, leaving them with less endurance and ability to effectively work in synchronization and less strength and stamina to support weight or keep the upright body system [3]. The elderly have limited capacity and endurance for training, contributing to unhealthy lifestyles that may lead to muscular weakening and atrophy and further add to low stamina and movement [4]. These populations are more likely to experience disrupted or impaired balance, gait style, and lack of bodily movement coordination, which may cause falls or phobias [5].

Medical practitioners often use the word "balance" across many therapeutic disciplines. Stability and postural control are two more principles that are commonly employed in connection with balance. The capacity of the physiological systems to keep the centre of mass within the base of support during static and dynamic postures and to govern physical alignment in an upright position is known as balance [6]. Impaired balance has a wide range of effects that can harm physical functionality. Falls linked to poor credit are believed to be more fatal than any other result, as falls are the leading cause of illness in the older population [7]. Balance dysfunction is one of the main reasons for less mobility and postural control. It has an effect on balance control in daily life and walking after discharge in community seniors [8]. The treatment of falls and the resulting morbidities necessitates the involvement of a large number of medical personnel. It may result in lengthier therapy sessions and additional work for the rehabilitation staff, which older people in the community typically do not pursue [9]. Balance issues are increasingly treated with the help of rehabilitation centres and technologies that can evaluate and mimic real-world conditions.

The foundation of virtual reality technology is a computer environment that enables users to produce graphic, auditory, and haptic insight and has an immersive element in a three-dimensional visual world designed to give users the impression that they are in a natural setting. This technology creates a virtual world with autonomy, interaction, and a sense of being. Patients who feel inadequate or have motor issues may use it throughout their rehabilitation program to get outcomes [10]. Virtual reality (VR) is a new and evolving technology that blends the liberty, engagement, and awareness of VR technology with rehabilitation therapy [11]. The definition of virtual reality is "an improved human-computer interaction mode that enables people to engage traditionally with a computer-based environment for instruction and full immersion" [12]. Exercise with the Wii Fit is safe for healthy older individuals [13]. Wii Fit training could help older adults balance, although this is debatable. The paper discusses the effect of Nintendo Wii Fit training on balance concerns in senior citizens.

## Review

Nintendo Wii Fit

Virtual reality (VR) is a technique with several applications that uses a variety of hardware, software, and equipment. It has been used in therapeutic interventions, rehabilitation, research, instruction, and evaluation. A review of the literature shows how much VR interests academics and medical professionals. This rise may be attributable to the development and acceptance of this technology, which has made digital media accessible to professionals and thus directly impacted the studies that employ this methodology. Nintendo Wii treatment (NWT) is among neurorehabilitation's most popular non-immersive virtual reality tools. The Nintendo Wii was the most frequently mentioned video game console in articles evaluated by search engines such as PubMed and Google Scholar. The Nintendo Wii is a simple technology for the therapist to use that the patient can use on their own. Unlike traditional video game systems like PlayStation, Kinect, or Xbox, Nintendo calls for more active engagement and movement [14]. Users of the Nintendo Wii can interact with a virtual world on display by using a handled controller or joystick; with the Wii Balance Board, they can use a force platform [15]. A virtual rehabilitation programme may be carried out using the Nintendo Wii, Wii Balance Board, and Wii Remote. A motion-detecting technology and sensors that respond to variations in direction, speed, and acceleration are used by the Wii Remote, a wireless controller, to interact with the player [16]. The Wii Balance Board is a small board that detects movements in the pressure centre and analyses the weight and force imparted to it, allowing balance training. The Wii Balance Board (WBB) platform, used for Wii Fit games, has four pressure sensors. While the player is standing, this platform instantly shows the position of the feet, the distribution of weight, and the shift of the centre of mass [17]. The Nintendo Wii is simple to include in a healthcare setting.

Additionally, it has been shown to deliver outcomes on par with gold-standard pressure platforms [18]. Although mirror neurons play a significant role due to the multimodal stimulation NWT provides, the benefits of NWT are primarily attributable to neuroplasticity and motor learning. According to several neuroimaging studies, using virtual reality tools for neurorehabilitation may encourage patients' brain plasticity and cortical remodeling. Based on the specific tasks, repetitions, intensity, and multisensory connections that NWT promotes, it may be possible to instigate activity-dependent brain plasticity. The exercises accessible in the various games of the NWT may adhere to the fundamental principles of neuroplasticity and motor learning [18]. Interactive exergaming entails multiple mental and physical activities with real-time user interaction provided by biofeedback technology [19]. Nintendo's biofeedback technology creates various training environments and task protocols as needed, resulting in more timely intervention completion than traditional physical therapy. Since video game systems are less expensive than designing specially made rehabilitation equipment, they are becoming more and more popular among those receiving biological treatment [20]. By adjusting the centre of pressure on the balancing board, the exerciser may move a fictitious avatar in the videogame while standing on it. The exerciser receives feedback from the game in both the visual and audible realms. This technique enables joint mobility, muscular power, and postural ergonomics training, combining pleasure with physical activity for all ages. The Nintendo Wii Fit Balance Board can be considered a portable and affordable posturography system to track advancements or deterioration in one's postural stability. Exergames can be a great alternative to indoor exercise and offer older adults a safer practice than outdoor exercise, even though they wouldn't replace physical activity. To encourage more senior players to expend more effort and continue playing, exergame designers should provide various difficulty levels and challenges to the games they create [21].

Postural balance control training using Nintendo Wii

With the emergence of commercial, off-the-shelf consoles and games over the past ten years, VR technology has rapidly increased. Although these systems were created for recreation, they have become a way to promote fitness games (also known as exergames) among the general public. Off-the-shelf systems are becoming increasingly popular as therapeutic modalities for motor and cognitive rehabilitation patients with various orthopaedic and neurological disorders since they are significantly less expensive (the cost of the setup in India is Rs. 48,538 or approximately USD 590.35) than custom-developed rehabilitation equipment. Among the most well-known commercial systems, the Nintendo Wii is the most commonly used for rehabilitation [21]. The major contributing factor in balance dysfunction or an unstable posture results from defects in integrating the multimodal data from the body's various systems, mainly the vision, proprioception, and vestibular systems. With Wiimote's three-dimensional accelerometer technology, which records arm motions, players can be encouraged to imitate actions carried out in real life, for instance. A gadget called the Wii Balance Board (BB) can track changes in a person's centre of pressure. Both peripheral devices can be utilised in games that test a player's ability to maintain balance while giving suitable visual and audio feedback on that performance [22]. Exergaming, active gaming, or game-based exercise are frequently used to describe the combination of training and gaming. Exercise helps older persons maintain their balance and reduces their risk of falling. But among older persons, participation in conventional exercises often declines with age. Therefore, to enhance balance in this group, new methods for exercising that are simple to use, appeal to older persons to maintain high levels of compliance, and activate multimodal systems and muscular power are required [23]. Exergaming is a cutting-edge approach to fitness training and rehabilitation. A gaming console, a balance board, and a wireless handheld pointing device with built-in sensors are all included with Wii Fit. The Wii Fit games encourage players to achieve a particular ideal body position. They are notified when they accomplish a point representation of their centre of gravity. The Wii Fit balance games make use of four fundamental movements: balancing on two legs, leaning forward, backward, and side to side; twisting the hips both clockwise and counterclockwise, and moving the body in time with a beat. A virtual reality programme that focuses on fitness can be employed to enhance postural stability. For efficient postural awareness, stability training, and sensory integration training for balance, the Nintendo Wii Fit and a balancing board are used. Physical therapists and doctors from various medical specialities frequently use Wii Fit in their clinical practice. Wii BB is multifunctional as it measures body weight, body mass index, and calories expended and detects deviations in posture [24].

Discussion

While exploring the benefits of exergaming in geriatrics, a recent study found that their participation improves living conditions. These also led to significant physical and intellectual improvements [25]. While playing these exergames, there is a requirement to execute coordinated movements. Strength and balance training had a more effective clinical impact on balance in geriatric females than virtual reality-based exercise [26]. A randomised control trial (RCT) conducted on the senior population in the community using game-based virtual reality concluded that there was a substantial improvement in the strength of the lower limbs and functional capacity. Training using Nintendo Wii might lead to maximum adherence to the community or home-established settings since the participant motivation levels are inflated [27]. In preceding studies, unescorted Wii Fit training improved balance. However, the impact was significant when the Wii Fit training was combined with conventional therapy. Improvements in balance using Wii Fit were limited to a certain extent in the geriatric population; it might be effective for those without exposure to physiotherapy [28].

According to a self-report, Wii Fit games are fun exercises. Because participants in the research continue to use it freely as an activity, Wii Fit balancing games have fostered and maintained exercise engagement among them; they have become a sustainable program. The Wii Fit therapy resulted in minor balance improvements [29]. The virtual reality training enhanced the respondents' balance ability with and without visual input. Due to issues with visual input, postural stability and harmony in the elderly may be compromised. Because of age-related vision loss, the subjects' reliance on visual information for balance reduces. Eyesight decline did not appear to influence balance impairment substantially in this investigation. Regular use results in a noticeable improvement in balance and a decrease in the danger of falling [30]. According to Ayesha Afridi et al.'s comprehensive study, Nintendo Wii Fit is an efficacious virtual reality strategy for the training of balance in a healthy senior population without any neurodegenerative or cognition problems. This technique takes less time for each session than typical physical therapy. It's also user-friendly, engaging, affordable, and administered at home [31]. The researchers should incorporate a larger sample size to validate the efficacy of such an intervention. Table 1 shows the summary of selected articles.

**Table 1:** Summary of the selected review of the literature.

Sr. No.	Author and year	Population	Intervention	Conclusion
1	Karasu AU et al. (2018) [32]	23 stroke patients	Nintendo Wii Fit and conventional physiotherapy	Virtual reality activities on the Nintendo Wii system may be a helpful supplementary therapy for stroke patients receiving standard treatment.
2	Fakro MA, et al (2020) [33]	88 older adults	A 40-minute session of standing balance with the "Table Tilt" game.	In this study done in Lebenon, it was found that Wii Fit balance training is reliable for enhancing older people's dynamic and static balance.
3	Cimino V et al. [34]	36 multiple sclerosis patients	Twenty individuals engaged in twenty separate Wii Fit Plus balancing training sessions.	According to the study, individuals with multiple sclerosis who underwent balance rehabilitation training on a Nintendo Wii balance board substantially reduced several postural sway indices.
4	Kannan L et al. (2019) [35]	24 people with chronic stroke	Hemiparetic, ambulatory PwCS completed six weeks of high-intensity, tapering balance training.	In clinical stroke rehabilitation settings, the use of cognitive-motor games has the potential to improve cognitive function and balance.
5	Afridi A et al. (2018) [36]	16 adults older than 60 years	All patients received the recommended Wii Fit Plus training, which included the games.	Wii Fit Plus training successfully enhanced older people's dynamic balance and mobility. Large-scale trials should be used to examine this further.
6	Khushnood Ket al (2021) [37]	90 older patients	The patients participated in 30-minute sessions of exercise gaming twice a week while the controls did balancing exercises.	Compared to workouts, exergaming helps the elderly with several gait-related issues.
7	Yoon-Kyun Shin Jung-Ah Kwon et al. (2022) [38]	41 hemiplegic patients	Traditional balance instruction from an occupational therapist and Nintendo Wii Fit balance training under supervision.	Wii Fit balance training helped hemiplegic patients overcome the unequal weight distribution on the afflicted side while improving their quality of life.
8.	Gomes GC et al. (2018) [39]	30 older adults	Participants played five of the ten games, giving each game two attempts.	Using Nintendo Wii Fit Plus enhanced frail older persons' postural control and gait and was practicable, acceptable, and safe.

## Conclusions

We conclude that Wii Fit physiotherapy is a successful virtual reality method for balance training in healthy older people as well as patients with neuro and cognitive dysfunctions. It can also be supplied at home and is user-friendly, attractive, and economical. Balance can be improved in this population with success using Wii Fit exercise games. The current study demonstrates that including community-dwelling older people in a Nintendo Wii-based training programme is feasible and may benefit their balancing abilities. As a result, such programmes might be used in place of more traditional kinds of exercise focused on strengthening and balance control. Better planned, systematic, controlled trials with adequate power, follow-up evaluations, and more systematic procedures and outcome measurements are needed before conclusive comments about the possibility of a Wii-based intervention as a safe and effective home-based treatment for community-dwelling older adults can be made.
